# Comparison of *Brachyspira hyodysenteriae* Isolates Recovered from Pigs in Apparently Healthy Multiplier Herds with Isolates from Herds with Swine Dysentery

**DOI:** 10.1371/journal.pone.0160362

**Published:** 2016-08-04

**Authors:** Tom La, Judith Rohde, Nyree Dale Phillips, David J. Hampson

**Affiliations:** 1 School of Veterinary and Life Sciences, Murdoch University, Murdoch, Western Australia 6150, Australia; 2 Institute for Microbiology, University of Veterinary Medicine, Hannover, Germany; Animal and Plant Health Agency, UNITED KINGDOM

## Abstract

Swine dysentery (SD) is a mucohaemorrhagic colitis of grower/finisher pigs classically resulting from infection by the anaerobic intestinal spirochaete *Brachyspira hyodysenteriae*. This study aimed to determine whether *B*. *hyodysenteriae* isolates from pigs in three healthy German multiplier herds supplying gilts to other farms differed from isolates from nine German production herds with SD. Isolates were subjected to whole genomic sequencing, and *in silico* multilocus sequence typing showed that those from the three multiplier herds were of previously undescribed sequence types (ST132, ST133 and ST134), with all isolates from the same herd having the same ST. All isolates were examined for the presence of 332 genes encoding predicted virulence or virulence lifestyle associated factors, and these were well conserved. Isolates from one multiplier herd were atypical in being weakly haemolytic: they had 10 amino acid substitutions in the haemolysin III protein and five in the haemolysin activation protein compared to reference strain WA1, and had a disruption in the promoter site of the *hlyA* gene. These changes likely contribute to the weakly haemolytic phenotype and putative lack of virulence. These same isolates also had nine base pair insertions in the iron metabolism genes *bitB* and *bitC* and lacked five of six plasmid genes that previously have been associated with colonisation. Other overall differences between isolates from the different herds were in genes from three of five outer membrane proteins, which were not found in all the isolates, and in members of a block of six plasmid genes. Isolates from three herds with SD had all six plasmid genes, while isolates lacking some of these genes were found in the three healthy herds—but also in isolates from six herds with SD. Other differences in genes of unknown function or in gene expression may contribute to variation in virulence; alternatively, superior husbandry and better general health may have made pigs in the two multiplier herds colonised by “typical” strongly haemolytic isolates less susceptible to disease expression.

## Introduction

The intestinal spirochaete *Brachyspira hyodysenteriae* is the classical causative agent of swine dysentery (SD), a disease typically associated with severe colitis and bloody mucoid diarrhoea mainly seen in grower and finisher pigs [1]. Two related species, *Brachyspira suanatina* and “*Brachyspira hampsonii*”, also have been shown to occur in pigs and to cause SD on occasion [2,3]. SD is a common disease that occurs in all pig rearing countries, and it can cause major economic losses in rearing herds due to reduced production, mortalities and costs of treatment. In addition the disease can represent an animal welfare issue if it is not adequately controlled. The occurrence of *B*. *hyodysenteriae* in breeding and multiplier herds that supply improved genetic stock to production herds also can cause major disruptions to the industry by impeding the trade and movement of pigs.

Although infection with *B*. *hyodysenteriae* can lead to typical SD, isolates of the spirochaete also have been recovered from apparently healthy herds [4–6]. A lack of disease in a colonised herd could be associated with the presence of *B*. *hyodysenteriae* strains with reduced virulence [7], the use of an effective medication regimen, and/or the occurrence of diet-related changes in physical or microbiological conditions in the large intestine that make the environment unfavourable for spirochaete colonisation [8–12].

Concern about the presence of the spirochaetes in breeding and multiplier herds could be ameliorated if there was an easy and reliable way to recognise strains with low virulence potential that are unlikely to cause disease if transferred to production herds. Experimental infection of pigs with such strains can give an indication of their virulence potential [4–6,13], but this procedure is expensive and complicated, and as a result is not practical for routine use. Alternatively, development of methods for recognition of attributes of the strain that would render them less likely to cause disease would be highly useful. A number of potential virulence or virulence lifestyle factors promoting colonisation have been described in *B*. *hyodysenteriae* [14,15]. These include haemolysins, phospholipases and lipooligosaccharide (virulence factors), and those associated with chemotaxis, motility, accessory factors for substrate utilization, iron binding, aerotolerance, and cell surface lipoproteins (virulence lifestyle factors). In addition a block of six genes of uncertain function on the ~36 kB plasmid have been suggested to be virulence lifestyle factors, promoting colonisation [16,17]. Strains lacking virulence lifestyle genes may be less able to colonise, or to colonise to critical numbers, whilst those lacking genes associated with tissue damage may not be able to cause typical lesions in colonised pigs.

This study compared *B*. *hyodysenteriae* isolates from three apparently healthy German multiplier herds to isolates recovered from pigs with SD in production herds in the same country. The aim was to determine whether isolates from the multiplier herds had differences that might explain their apparent lack of virulence. No consistent genomic differences were found that might account for differences in disease expression by the isolates from the three multiplier herds, except in the case of weakly haemolytic isolates from one multiplier herd that had a disruption in the promoter region for the haemolysin gene *hlyA*, as well as changes in the haemolysin III and haemolysin activation proteins, and in the iron metabolism genes *bitB* and *bitC*. None of the isolates from the three multiplier herds possessed all six of the block of plasmid genes, but isolates with a similar lack of plasmid genes also were found in some herds with disease, indicating that even if these genes do enhance colonisation potential they are not essential for disease production.

## Materials and Methods

### Source of *Brachyspira hyodysenteriae* isolates

This study utilised 23 *B*. *hyodysenteriae* isolates obtained from the culture collection at the University of Veterinary Medicine, Hannover. The isolates originally had been recovered from diagnostic faecal samples received from 12 German pig herds, submitted in 2014. Thirteen isolates were from three healthy multiplier herds A, B and C (n = 6, 4 and 3 isolates, respectively) and were recovered during routine screening of faecal samples from healthy pigs to confirm the ongoing high health status of the herds. These herds supplied genetically improved stock to production herds. They did not regularly use antimicrobials that could mask the presence of disease in the herds. The other ten isolates were submitted from nine production herds that had a clinical history of SD, but that were not directly linked to the multiplier herds (Table 1).

**Table 1:** Origin, herd disease status, sequence type, strength of haemolysis and plasmid gene (ORF) profiles of the 23 *B*. *hyodysenteriae* isolates tested.

Isolate	Herd	Disease	ST^a^	Haemolysis^b^	Block of plasmid genes^c^					
					ORF11	ORF12	ORF13	ORF14	ORF15	ORF16
JR1	A	no SD	132	S	N	N	P	P	P	P
JR2	A	no SD	132	S	N	N	P	P	P	P
JR3	A	no SD	132	S	N	N	P	P	P	P
JR4	A	no SD	132	S	N	N	P	P	P	P
JR5	A	no SD	132	S	N	N	P	P	P	P
JR6	A	no SD	132	S	N	N	P	P	P	P
JR7	B	no SD	133	S	N	N	N	N	P	N
JR8	B	no SD	133	S	N	N	N	N	P	N
JR9	B	no SD	133	S	N	N	N	N	P	N
JR10	B	no SD	133	S	N	N	N	N	P	N
JR11	C	no SD	134	W	N	N	N	N	P	N
JR12	C	no SD	134	W	N	N	N	N	P	N
JR13	C	no SD	134	W	N	N	N	N	P	N
JR36	D	SD	138	S	P	P	P	P	P	P
JR37	D	SD	138	S	P	P	P	P	P	P
JR25	E	SD	**118**	S	P	P	P	P	P	P
JR27	F	SD	**104**	S	P	P	P	P	P	P
JR21	G	SD	**112**	S	N	N	P	P	P	P
JR23	H	SD	137	S	N	N	N	N	P	N
JR19	I	SD	**52**	S	N	N	N	N	P	N
JR20	J	SD	136	S	N	N	N	N	N	N
JR24	K	SD	**120**	S	N	N	N	N	N	N
JR38	L	SD	**120**	S	N	N	N	N	N	N

^a^ ST, sequence type. Previously described STs are marked in bold. See Table 2 for details.

^b^ S, strong haemolysis; W, weak haemolysis

^c^ The six plasmid ORFs respectively encode three Radical SAM domain proteins; glycosyl transferase, group 1-like protein;

NAD dependant epimerase; and dTDP-4-dehydrorhamnose 3,5-epimerase (*C*), respectively. N; absent, P; present

Unexpectedly, *B*. *hyodysenteriae* was isolated from multiplier herd A in 2014 during routine health screening. As the herd supplied pigs to other herds, an eradication program was performed in which the herd was depopulated for six weeks before being repopulated from the same high-health-status herd it had used previously as a source of animals. A thorough clean up, disinfection and pest control programme was undertaken by a professional company during the depopulated period; subsequently, routine sampling indicated that the farm was free of *B*. *hyodysenteriae*. Six months later the spirochaete again was isolated from faecal samples during routine screening. Isolates from before (n = 1) and after (n = 5) the eradication attempt were available for examination. A similar apparently subclinical colonisation subsequently was found in multiplier herd B located approximately 30 kilometres from herd A, and which received additional testing because it received piglets from the same source as herd A. As well as *B*. *hyodysenteriae*, an isolate of "*B*. *hampsonii*" that subsequently was identified as genetic group III in multilocus sequence typing [18] was recovered during surveillance. Herd C was a closed herd that had not receive any live pigs for 15 years, and was located in a different region of Germany to herds A and B. None of the three herds had direct contact with each other.

### Culture and identification

Diagnostic faecal samples and colon contents were cultured on selective Trypticase Soy agar (TSA) supplemented with 0.1% yeast extract, 6 μg/ml vancomycin, 6.25 μg/ml colistin, 12.5 μg/ml rifampicin, 15.25 μg/ml spiramycin, 200 μg/ml spectinomycin, and 5% bovine blood [19], and on Columbia blood agar (CBA), all supplied by Oxoid, Wesel, Germany, and were incubated anaerobically in an AnaeroJar with an AnaeroGen generator (Oxoid) at 42°C for six days. Suspected brachyspiral growth was confirmed by phase contrast microscopy and identified to the species level using *nox*-RFLP as described previously [20–22]. Isolates identified as *B*. *hyodysenteriae* subsequently had their identity confirmed using a *B*. *hyodysenteriae* species-specific PCR [23] and *nox*-gene sequencing using the same primers as for *nox*-RFLP. Interpretation of sequencing results was based on CLSI guideline MM18-A [24]. Prior to genomic sequencing the identity of the *B*. *hyodysenteriae* isolates again was independently confirmed using a modified *nox*-based PCR [25].

### Strength of haemolysis

The strength of beta haemolysis around brachyspiral growth on the isolation plates was recorded for all isolates. The strength of haemolysis for all isolates was tested again both on TSA with 10% bovine blood and on CBA with 5% ovine blood, where a cut was made in the agar during inoculation to enhance any haemolysis (the “ring phenomenon” test). Haemolysin production also was induced for up to two hours from resting cells using RNA-core type XI-C (Sigma-Aldrich, St. Louis, USA), as previously described [26]. Briefly, spirochaete cells in exponential growth were harvested from TSA plates after 5 days growth. The cells were washed three times with sterile phosphate buffered saline (PBS) before being resuspended in PBS supplemented with 2mM glucose, 2mM MgSO_4_ and 0.2% (w/v) RNA-core. Spirochaetes were counted in a haemocytometer chamber viewed with a phase contrast microscope, numbers were adjusted to approximately 1 x 10^6^ cells per ml with the same buffer, and 2ml of cell suspension was incubated at 37°C. A sample of the cells (0.5ml) was taken before incubation and after 30, 60 and 120 minutes of incubation at 37°C. The collected samples were immediately placed on ice and the supernatant collected after centrifugation at 10,000 *g* for 10 minutes. Undiluted supernatants (50μl) were added to 50μl of washed sheep red blood cells (RBC) that were diluted 100-fold to approximately 5 x 10^7^ erythrocytes per ml in a 96-well V-bottom plate. Haemolysis was allowed to occur at 37°C for one hour. *B*. *hyodysenteriae* strain WA1 was included as the strong haemolysis control and *Brachyspira innocens* strain B256 was used as the weak haemolysis control. A positive control for the assay comprising 50μl of sterile distilled water and a negative control comprising 50μl sterile PBS also were included. Haemolysis was visually examined, with positive haemolysis identified as the absence of settled RBC in the bottom of the well, similar to that observed for the positive control. Negative (weak) haemolysis was identified by the presence of settled RBC at the bottom of the well, comparable to results for the weakly haemolytic *B*. *innocens* control. All samples were tested in triplicate.

### Whole genomic sequencing

Bacterial DNA was extracted from five-day old cultures of *B*. *hyodysenteriae* using the DNeasy Blood and Tissue Kit (Qiagen, Hilden, Germany). Genome sequences of *B*. *hyodysenteriae* strains were generated on an Illumina MiSeq using v3 chemistry and 300 base pair (bp) paired-end reads using dual indexed Nextera XT libraries. The mean insert size was around 250–300 bp and the sequencing was performed at 70x depth of coverage. MiSeq reads were paired using the inward pointing orientation typical for Illumina paired end sequencing. *De novo* assembly was performed using Geneious R9 (Biomatters Ltd, Auckland, New Zealand) using a high sensitivity setting with reads not trimmed prior to assembly. Assembled contiguous sequences were mapped to the genome sequence of *B*. *hyodysenteriae* strain WA1 (accession number NC_012225) using CONTIGuator [27] with a Blast E-value of 1e-20. The assembled contigs were deposited at GenBank as a whole genome sequencing (WGS) project with BioProject identification PRJNA326554.

### *In silico* multilocus sequence typing (MLST)

MLST was conducted using the previously described scheme [28], except that for each locus the first allele sequence for the *B*. *hyodysenteriae* MLST locus was used to identify the allele sequences for each sequenced *B*. *hyodysenteriae* strain using the BlastN function of the Geneious R9 software. Allele designations for each locus then were obtained by a query search from the PubMLST website. New sequence type (ST) designations were assigned and deposited at the PubMLST site (http://pubmlst.org/brachyspira/), and existing data concerning the STs of other *B*. *hyodysenteriae* isolates was downloaded.

The MLST allele sequences for each *B*. *hyodysenteriae* strain were concatenated in the order of *adh*-*alp*-*est*-*gdh*-*glp*K-*pgm*-*thi* and the concatenated sequences aligned with ClustalW. Dendograms were constructed from concatenated allelic sequences using the Unweighted-Pair Group Method with Arithmetic Mean (UPGMA) method with 1000 bootstrap replicates in MEGA6 [29]. Minimum spanning trees (MST) were constructed from the data matrix of allelic mismatches using the UPGMA method with 1000 bootstrap replicates using the PHYLOViZ software [30].

### Virulence and virulence lifestyle genes

Gene sequences for the putative virulence and lifestyle genes of *B*. *hyodysenteriae* strain WA1 were obtained from previous publications [14,15]. A total of 332 genes that have been associated with virulence or virulence life style factors in *B*. *hyodysenteriae* were examined in detail (S1 Table). These included the 11 genes that previously were investigated by PCR in 121 German isolates from pigs with SD [31]. These were genes for inner membrane (*clpX*) and outer membrane proteins (OMPs: *bhlp16*, *bhlp17*.*6*, *bhlp29*.*7*, *bhmp39f*, *bhmp39h*) that potentially could be involved in adhesion or interactions with host cells, haemolysins (*hlyA/ACP*, *tlyA*), iron metabolism (*ftnA*, *bitC*), and aerotolerance (*nox*). Additional haemolysin genes *tlyB* and *tlyC*, and those predicted to encode haemolysin III, haemolysin activation protein, haemolysin and haemolysin channel protein also were investigated [14,15], as were *bitA* and *bitB*, genes associated with iron metabolism. Other genes examined included five *rfb* genes associated with rhamnose biosynthesis, which presumably are involved in O-antigen production, and members of a block of six plasmid-encoded genes that recently have been described as putative virulence-associated genes in *B*. *hyodysenteriae* [17], one of which is a second copy of an *rfbC* gene mentioned above.

Each *B*. *hyodysenteriae* genome sequence was searched using the BlastN function of Geneious R9 software for the presence of the putative virulence-associated genes. Sequences with a maximum E-value less than 1e-50 and having greater than 80% sequence length were extracted for further analysis. Homologous gene sequences were translated in Geneious R9 and both nucleotide and deduce amino acid sequences were aligned to the *B*. *hyodysenteriae* strain WA1 homolog using the MUSCLE alignment tool from Geneious R9.

The nucleotide and translated amino acid sequences of the virulence-associated genes also were examined to determine whether there were any consistent differences amongst the isolates from herds with and without disease.

### Investigation of the genomic environment around the haemolysin genes

Genomic regions directly adjacent to the coding sequence of the eight haemolysin-associated genes were examined using the MUSCLE alignment tool from the Geneious R9 software to identify potential differences in the promoter regions amongst the isolates.

## Results

### Spirochaete isolation

Spirochaetal growth was recorded as being sparse or occasionally moderate from faecal samples from the three multiplier herds, whereas it was heavy for all the samples recovered from the nine production herds with SD. Twenty colon samples that were examined from pigs from herd B all showed chronic lymphoplasmacytic colitis with hyperplasia of the gut-associated lymphoid tissue. Samples from one animal also showed depletion of goblet cells, and *Brachyspira murdochii* was isolated from this sample. A heavy growth of “*B*. *hampsonii*” was recovered from one colon sample, and heavy and sparse growths of *B*. *hyodysenteriae* were recovered from two other colon samples. Three sows from herd C were necropsied and no gross or histological findings consistent with SD were observed, although moderate or heavy growth of *B*. *hyodysenteriae* was recovered from the colons. None of the clients receiving pigs from the three multiplier herds prior to them ceasing supplying stock reported subsequent occurrence of SD.

### Genome assembly

Sequencing depth of coverage of the Illumina 300 base paired end reads and assembly statistics for the 23 strains of *B*. *hyodysenteriae* is shown in S2 Table. The genome size of all strains was approximately 3 Mb (3,172,065 to 3,622,295 bp), which is similar to the *B*. *hyodysenteriae* reference strain WA1. The GC content was around 27.1%, which also is in agreement with values for the reference strain [14,15].

### Mapping to reference strain WA1 genome and plasmid

The results for mapping the assembled contiguous sequences to the reference strain WA1 genome is shown in S3 Table. All strains had a genome sequence alignment coverage between 2.28% and 18.2% compared to the WA1 genome. Of the mapped sequences, the similarity to the WA1 genome ranged from 94.04% to 96.98%.

### Multilocus sequence typing

**Fig 1:**
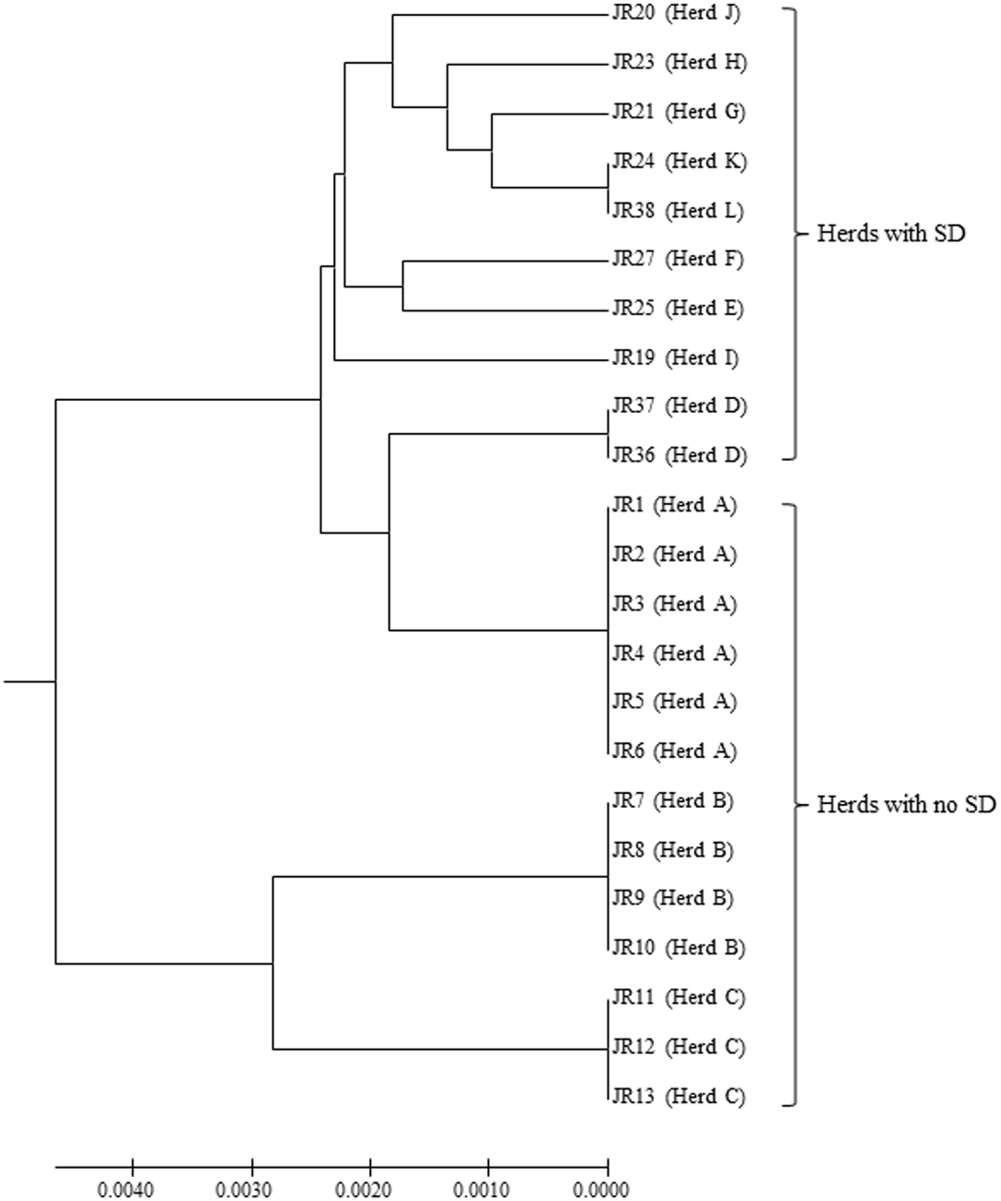
MSLT dendrogram showing relationships between the 11 STs of German *B*. *hyodysenteriae* isolates from this study. The dendrogram was constructed from concatenated allelic sequences using the Unweighted-Pair Group Method with Arithmetic Mean (UPGMA) method with 1000 bootstrap replicates in MEGA6. Isolates originating from pig herds showing clinical signs of disease and the three asymptomatic pig herds are indicated. Evolutionary distances were computed using the Maximum Composite Likelihood method and are shown as the number of base substitutions per site.

The sequence types (STs) for the *B*. *hyodysenteriae* isolates are shown in Table 1, and the MLST dendrogram for these STs is shown in Fig 1. The 23 German isolates were assigned to 11 STs (Fig 1). The isolates from multiplier herds B and C were more closely related to each other than to those in the other nine STs, originating from multiplier herd A and the production herds. The isolates from multiplier herds A, B and C were of previously undescribed STs (ST132, ST133 and ST134, respectively), and all isolates from the same herd had the same ST. The isolates recovered from herd A before and after the eradication programme had the same ST (ST132), suggesting that the same strain had re-emerged. The two isolates from herd D also shared a common ST (ST138) and the single isolates from herds K and L both were of ST120, whilst all other isolates from the different herds had different STs. Three of the STs from the nine herds with SD were newly described whilst the other five had STs that previously have been described. The latter included strains from Germany (ST52, ST112, ST118 and ST120), Belgium and Italy (ST52), and the USA (ST104), recovered in the 1990s, 2000s and 2010s (Table 2). Weakly haemolytic strain D28 from Belgium belonged to a different ST (ST172) from the isolates in the current study [32].

**Table 2:** Name, origin and date of isolation for previous porcine isolates that had the same sequence type (ST) as those in this study.

ST	Isolate name	Country of origin	Year isolated
52	A5677/96	Germany	1996
52	Be45	Belgium	1990s
52	T20	Germany	1990s
52	30i	Italy	2005
52	227	Italy	2009
104	NM62	USA	2012
104	NM35	USA	2012
104	NM63	USA	2012
112	V0779/05	Germany	2005
118	V2641/04	Germany	2004
120	V332/13	Germany	2013

**Fig 2:**
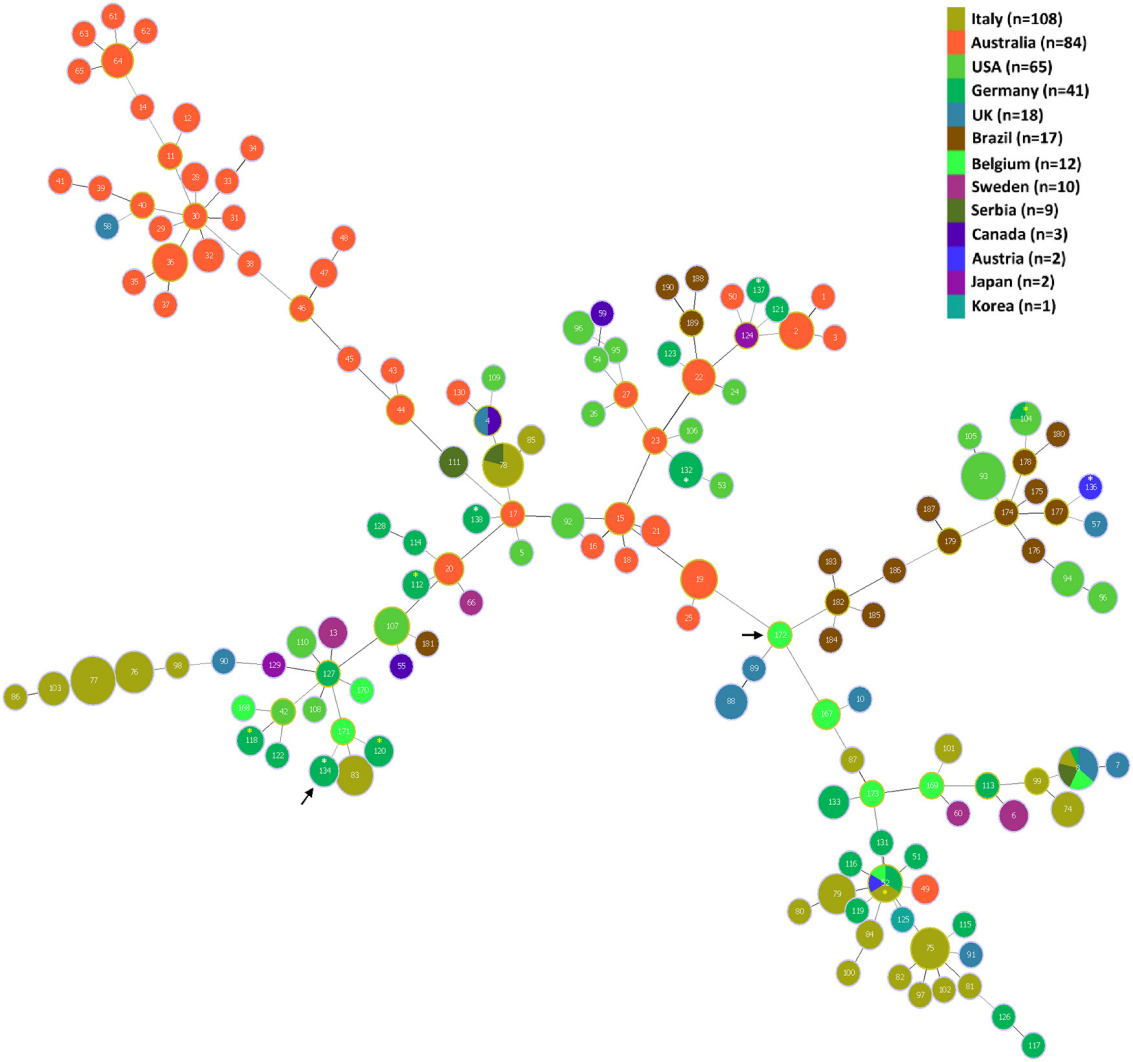
Minimum Spanning Tree showing relationships of the *B*. *hyodysenteriae* isolates from this study (marked with an asterisk) and 349 previously described isolates that were obtained from PubMLST. The isolates shown originated from Australia, Austria, Belgium, Brazil, Canada, Germany, Italy, Japan, Korea, Serbia, Sweden, the UK and the USA. In the MST, each node represents a different ST (labelled) and the colour represents the country of origin. The size of the node indicates the number of strains having the same ST. The STs containing the isolates from the current study are indicated by an asterisk, which are coloured white for the new STs and yellow for previously described STs. The STs of the weakly haemolytic isolates from herd C in the current study (ST134) and of weakly haemolytic strain D28 from a previous study (ST172) [32] are shown with arrows.

A Minimum Spanning Tree showing the relationships of the STs to each other and to previously described STs is presented as Fig 2. Although the isolates from herds A, B and C belonged to newly described STs, they were closely related to other STs (see http://pubmlst.org/brachyspira/). For example, the ST of isolates from herd A (ST132) shared four MLST alleles with ST23 containing an Australian isolate from the 1980s, and four with ST53 containing a USA isolate from the 1970s; the ST of isolates from herd B (ST133) shared four alleles with ST87 containing an Italian isolate from 2006, but also shared three alleles with ST52 containing isolates from Germany, Belgium, Italy and Austria isolated between 1996 and 2009; the ST of isolates from herd C (ST134) shared three alleles with ST112 and with ST120 which included German isolates recovered in 2005 (ST112) and 2013 (ST120), and current isolates from herds G, and K and L respectively.

### Isolates and haemolysis

The isolates from herd C were all weakly haemolytic, whilst all other isolates were strongly haemolytic. When resting cells were induced in the presence of RNA-core, haemolytic activity was detected in the supernatant of the strongly haemolytic isolates after 30 minutes incubation whereas no haemolytic activity was detected in the supernatant of the weakly haemolytic isolates, even after 120 minutes incubation.

### Presence of genes encoding virulence and virulence lifestyle associated factors

In all cases isolates sharing an ST had the same virulence gene profiles and could be regarded as representing a single strain. Consequently the strain name used is that of the first isolate recovered from each herd (eg JR11 for herd C). Of the 332 genes investigated, 12 (3.8%) were absent in one or more isolates. The first six were membrane protein genes: *bhlp16* (which was absent from isolates with STs 133, 134, 112 and 137 from herds B, C, G and H respectively), *bhlp17*.*6* (which was only found in the isolate in ST137 from herd H), *bhmp39e* and *bhmp39f* (both genes absent from isolates with STs 133, 134 and 137 in herds B, C and H respectively), putative cysteine peptidase BHWA1_RS01825 (absent from isolates with STs 132, 120, 138, 118 and 137 in herds A, C, D, E, H and K respectively), and putative inner membrane protein BHWA1_RS02825 (absent from isolates with STs 132, 133, 134, 120, 138, 152 and 136 in herds A, B, C, D, I, J and K respectively).

In addition there were differences between isolates in members of the block of six plasmid genes (ORFs 11–16; Table 1). Isolates with STs 138, 118 and 104 from herds D, E and F (all with SD) had all six plasmid genes present; isolates with ST132 (no disease) and ST112 (with SD) lacked two genes (ORFs 11 and 12); isolates with STs 133 and 134 (no disease), and STs 137 and 52 (with SD) only had one of the genes present (ORF15); and isolates with STs 136 and 120 (with SD) had none of the six genes.

### Sequence similarity amongst the genes studied

Generally the between-strain comparisons of the sequences of the genes and their translated amino acid sequences showed that these were highly conserved, having greater than 90% similarity at the nucleotide level and greater than 92.1% similarity at the amino acid level.

#### Sequences of *bitB* and *bitC*

The predicted *bitB* and *bitC* sequences from the strains from herds C and I both had a 9-nucleotide insertion after position 90 which resulted in three amino acids inserted after residue 26 of the translated protein sequence. There also was a high degree of variation at positions 4 to 32 and positions 93 to 120 of the translated protein sequences. The BitB and BitC proteins both had 11 of 29 (37.9%) amino acid substitutions between positions 4 and 32, and 13 of 28 (46.4%) substitutions between positions 93 and 120. Outside these regions of variation the nucleotide and amino acid sequences of the two genes were well conserved. The *bitA* sequence was almost identical between strains, except in the region between base 104 and 392 where the similarity was 73% (211 out of 289 nucleotides). When translated the protein was 65.6% (63 out of 96 nucleotides) similar.

#### Sequences of haemolysis-associated genes

A summary of nucleotide and predicted amino acid substitutions in the eight haemolysis-associated genes from the weakly haemolytic strain J11 from herd C compared to strongly haemolytic reference strain WA1, and to weakly haemolytic *B*. *hyodysenteriae* strain D28 isolated from pigs in Belgium, as described by Mahu et al [32], is shown in Table 3. The greatest difference between strain J11 and WA1 occurred in the haemolysin III protein (10 amino acid substitutions), with the haemolysin activation protein having five substitutions. D28 had five different amino acid substitutions in the haemolysin III protein, and five amino acid substitutions in the haemolysin activation protein that were identical to those in strain J11. It is important to note that in the paper of Mahu et al [32] the labelling for haemolysin III and haemolysin activation protein in their Tables 2 and 3 and in their text appear to have been inadvertently transposed.

**Table 3:** Nucleotide and amino acid differences for eight haemolysis-associated genes between strongly haemolytic *B*. *hyodysenteriae* reference strain WA1, weakly haemolytic strain J11 from herd C, and previously described weakly haemolytic strain D28 [32].

Locus and gene name	Differences for J11 gene	Differences for J11 protein		Differences for D28 gene	Differences for D28 protein	
		Position	Substitution		Position	Substitution
BHWA1_RS12830: *hly*A	3 nt	18	serine → proline	none	none	none
BHWA1_RS01170: *tly*A	4 nt	51	valine → isoleucine	1 nt	501	glycine → cysteine
BHWA1_RS05965: *tly*B	6 nt	none	none	2 nt	384	alanine → threonine
BHWA1_RS06925: *tly*C	none	none	none	4 nt	none	none
BHWA1_RS02195: hemolysin III	61 nt	30	glycine → serine	44 nt	51	proline → serine
		47	threonine → isoleucine		56	valine → isoleucine
		49	valine → methionine		59	valine → leucine
		56	valine → isoleucine		82	leucine → isoleucine
		79	leucine → isoleucine		93	valine → isoleucine
		82	leucine → isoleucine			
		111	valine → isoleucine			
		114	leucine → proline			
		133	methionine → isoleucine			
		213	cysteine → alanine			
BHWA1_RS02885: hemolysin activation protein	72 nt	81	valine → isoleucine	63 nt	81	valine → isoleucine
		113	methionine → valine		113	methionine → valine
		164	glutamic acid → aspartic acid		164	glutamic acid → aspartic acid
		227	serine → threonine		227	serine → threonine
		265	valine → isoleucine		265	valine → isoleucine
BHWA1_RS09085: hemolysin III channel protein	13 nt	209	valine → isoleucine	12 nt	217	arginine → isoleucine
BHWA1_RS04705: hemolysin	21 nt	197	asparagine → aspartic acid			
		347	serine → glycine	not done		
		431	threonine → asparagine			

#### Promoter regions

**Fig 3:**
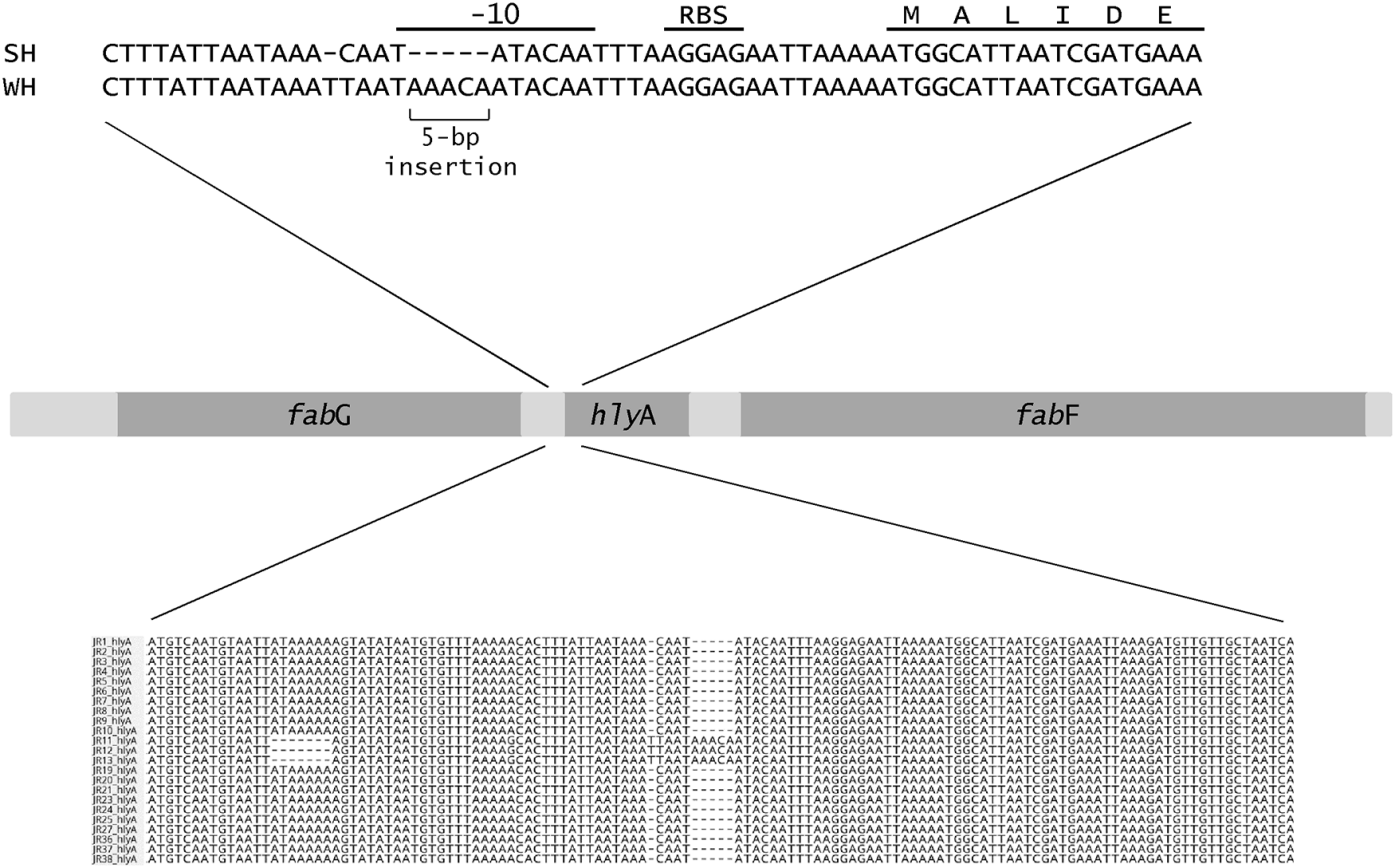
Position of the five-nucleotide insertion found in the promoter site of *hlyA* in the weakly haemolytic strain J11 from herd C. The ribosome binding site (RBS) and Pribnow box (-10 element) are indicated. Also note the seven base deletion further upstream in the weakly haemolytic isolates.

Analysis of the nucleotide sequences around all eight genes linked to haemolytic activity identified a five-nucleotide insertion in the -10 promoter element for the *hlyA* gene in the weakly haemolytic strain J11 from herd C (Fig 3). There was a nucleotide substitution (C to T) 28 bp upstream from the ATG start position, a single base insertion (T) 29 bp upstream from the start position, a substitution (A to G) 45 bp upstream and a seven base deletion starting 66 bp upstream. No changes in the promoter sites for the other genes were identified.

## Discussion

The first important finding in this study was the confirmation that each of the three multiplier herds was colonised by strains of *B*. *hyodysenteriae* that were not genetically atypical of the species (Fig 2). The second was that each of the multiplier herds had its own new strain of *B*. *hyodysenteriae*, and consequently that they were unlikely to have been infected from a common source. This was particularly important for herds A and B as they both received piglets from the same breeding herd. As the isolates from herd A that were recovered before and after the eradication program shared the same ST and virulence gene profile, the most likely explanation is that this is a single strain that persisted in the farm environment following the destocking and cleaning procedure.

It was unclear how long the subclinical colonisation had been present in the multiplier herds, but as they were found almost by chance they may have been present for some time—and this leads to an implication that other apparently healthy multiplier herds potentially could be similarly colonised, but remain unidentified. The herds were attended by expert veterinary consultants and had a routine of submitting normal faecal samples for examination every six months, as well as samples from grower and finisher pigs with diarrhoea. Unfortunately this screening regimen apparently is not sufficiently sensitive for detection, as initially only herd A was identified as being colonised through this route. A scenario of subclinical colonisation could have major consequences for the spread of infection through the German pig industry. Indeed, the problem is not limited to Germany as recently an infected Swiss multiplier herd was identified [33], as have similar apparently healthy herds in Australia [7,34]. The latter infected herds initially were detected through serological screening, with confirmation of colonisation being obtained by culturing the colonic walls of apparently healthy pigs at the abattoir. It seems likely that the problem of unrecognised colonisation also may occur in other pig rearing countries, but may not have been investigated adequately.

A potential explanation as to why the strains in the multiplier herds were not causing disease is that they were of reduced virulence compared to typical *B*. *hyodysenteriae* isolates, being either less able to colonise to numbers great enough to cause disease and/or be detected, and/or lacking virulence factors able to induce typical lesions [4,6,13,35]. Given that different strains (STs) were identified in the three multiplier herds, intuitively such a lack of virulence is less likely to be the case for all three herds. However, in support of a possible reduced colonisation potential was the finding that in all three herds only relatively low numbers of spirochaetes were recovered from the faeces, whereas high numbers were recovered from the production herds with disease. On the other hand spirochaete numbers in the colon tended to be higher than in the faeces. In relation to an ability to produce lesions, in herd B the diagnostic colonic samples that were examined displayed chronic lymphoplasmic colitis, which is not consistent with acute SD where pigs typically display mucohaemorrhagic colitis [1]. *B*. *murdochii* and “*B*. *hampsonii*” also were isolated from herd B, and potentially persistent colonisation by members of these species could contribute to a chronic lymphoplasmic colitis. In contrast, lesions were not found in three sows from herd C despite them having a heavy growth of *B*. *hyodysenteriae* in their colons. These findings are consistent with the isolates from these two herds potentially having different but relatively reduced virulence potential compared to isolates from the production herds. Strain J11 from herd C seemed to be particularly innocuous.

The isolates from the nine herds with SD belonged to three newly identified STs and to five that previously have been recorded in European countries or the USA (Table 2). Consequently these isolates also could be considered as being typical of those in circulation in herds with SD in Europe, and so should represent a good set to compare to the isolates from the three multiplier herds where disease was not seen. This comparison involved genes that previously have been associated with factors that may influence the ability to colonise (virulence life style genes) and those thought to encode factors that generate tissue damage (virulence genes) [31]. As it has proved very difficult to genetically manipulate *B*. *hyodysenteriae*, most of these associations between genes and their function have not been tested experimentally, and represent assumptions. Furthermore there are many genes of unknown function in the genome, and some of these also are likely to be important for disease production [14,15].

In a previous genomic analysis of 20 strains of *B*. *hyodysenteriae* substantial conservation was found in the presence and sequence of virulence-associated genes [15], and the same relative conservation was found amongst the isolates examined here.

Various studies have shown that lipooligosaccharide (LOS) extracted from *B*. *hyodysenteriae* has a variety of effects that can induce local inflammation and tissue damage that may contribute to the lesions of SD [36,37]; however, in this study there were no consistent differences between the German isolates in the number or distribution of *rfb* genes or others associated with LOS production. Previously *B*. *hyodysenteriae* isolates from pigs, ducks and rodents in Sweden also were shown to have conserved *rfb* genes [38]. Other attributes such as motility and chemotaxis are extremely important for the spirochaetes to allow them to colonise the large intestine [39,40], but again the genes associated with these processes were highly conserved in the strains investigated. The *bitABC* gene cluster potentially can influence the ability of the spirochaetes to bind iron, and hence alter their capacity to survive in the colon, but again these were conserved apart from in the isolates from herd C and isolate JR19 from herd I (with SD) which had insertions resulting in changes in their sequence. Unfortunately there is no specific information around the predicted active sites/binding sites of these proteins [41], so it is difficult to determine how important these changes are likely to be in relation to gene function.

The only substantial differences in distribution of putative virulence gene across the full range of isolates were found for some of the membrane proteins and lipoproteins, and in the block of six plasmid genes; however, no clear associations between these gene profiles and virulence phenotype were noted. These findings indicate that most of the previously described virulence-associated genes form part of the core genome of the species.

Differences in the presence of genes encoding outer membrane proteins in the various isolates may result from selection pressure exerted by the immune system. Compared to the profiles previously reported for other German isolates, a notable difference was that *bhlp29*.*7* was present in all isolates in the current work, whilst it was only amplified by PCR in around half the isolates in the previous study [31]. This has some practical implications because Bhlp29.7 (formerly BmpB) has been proposed for use as an antigen for a serological ELISA for detection of evidence of *B*. *hyodysenteriae* infection [42], and conservation and distribution of the protein across all strains is important for its use in detecting infected herds.

The greater variation in the block of plasmid genes between isolates compared to that found in most other genes was interesting, and may suggest that their conservation and function is less critical for spirochaete survival than other potential virulence lifestyle genes. The three STs with isolates possessing the full block of six plasmid genes came from herds with SD, and this is consistent with these genes potentially being involved in enhancing colonisation capacity. On the other hand, the isolates in two STs lacking all these genes also came from herds with SD. In the case of the latter herds it is possible that other isolates with a full set of plasmid genes also were present in lower numbers, but were not isolated and tested, or that there were other predisposing conditions in these herds that overcame any disadvantage arising from an absence of plasmid genes in these isolates, in terms of their capacity to colonise. The isolates from the three asymptomatic multiplier herds did lack members of the block of plasmid genes, but this was only ORF11 and ORF12 (encoding two of the three Radical SAM domain proteins) for isolates from herd A, whilst the isolates from herds B and C lacked all but ORF15 (encoding NAD dependant epimerase). To date the exact role of the individual plasmid genes is unknown, and any sub-classifications based on the number and identification of the absent genes and their functions have not been investigated experimentally. Overall, this study suggests that an absence of plasmid genes cannot be used to predict that a given strain of *B*. *hyodysenteriae* will not cause disease, even though strains lacking these genes may be less likely to produce disease.

Of the virulence factors, the haemolytic activity of *B*. *hyodysenteriae* is believed to be particularly important for lesion production, and eight genes encoding proteins predicted to be involved in haemolytic activity have been described [14,15,43,44]. The number and the sequences of all these genes generally were well conserved across the isolates in the current study. In relation to the weakly haemolytic strain J11 from herd C, the haemolysin III gene and protein were the most dissimilar from those of WA1 (Table 3), having10 amino acid substitutions that were predicted to occur in both transmembrane and topological domain regions of the channel-forming protein, based of the structure of yqfA in *Escherichia coli*. In comparison weakly haemolytic strain D28 from Belgian pigs [32] only had five substitutions in this protein, although potentially they also could influence function. Strain J11 from herd C also had five amino acid substitutions in the haemolysin activation protein, and it was very interesting that the same substitutions occurred in this protein in D28, even though the two strains were not otherwise closely related genetically. Other changes in haemolysin-associated genes in the strain from herd C were relatively minor, but included three amino acid substitutions in the haemolysin gene (not examined by Mahu et al [32]). Potentially any of these changes could affect the function of the proteins and might influence haemolytic activity, which in turn is likely to be linked to virulence. Unfortunately there have been no detailed functional/structural studies of the haemolysis-associated genes in *B*. *hyodysenteriae*, so it is difficult to predict how an amino acid change might influence function.

Besides gene distribution and sequence, the control of gene expression is likely to be critical in influencing the phenotype of the strains. This was seen in the case of *hlyA*, where although the gene was present in all strains examined, there was a disruption to the promoter region of *hlyA* in the strain from herd C (Fig 3). Induction of haemolytic activity in the presence of RNA-core has been associated with the production of HlyA [44], and this induction failed to occur with the weakly haemolytic strain. Haemolytic activity, particularly associated with HlyA, is considered integral to the pathogenesis of SD by causing disruptions to the colonic epithelium [44], and hence the disruption to the gene may be useful as a predictive marker for lack of virulence. This same strain from herd C also had substitutions in haemolysin III and haemolysin activation proteins that could influence its haemolytic properties, and it also had insertions in *bitB* and *bitC*, which potentially could affect iron binding. To further investigate the functional significance of these and other such genetic changes it will be important to undertake transcriptomic and proteomic analyses, to investigate gene function by using targeted gene mutagenesis and complementation experiments, and to undertake *in vitro* functional assays for iron binding, as well as other assays for chemotaxis, motility, and haemolysis. Cell or tissue cultures potentially also could be used to look for variation in cellular responses to different isolates [45,46].

Overall it seems likely that strain J11 from herd C (ST134) had a reduced virulence potential, since it was weakly haemolytic, had insertions in *bitB* and *bitC*, and lacked five of the block of six plasmid genes. Furthermore colonised sows did not show any colonic lesions. Ultimately it would be necessary to undertake experimental infection of pigs with such strains under controlled conditions to confirm their true virulence potential [4–6,13,35]. This process requires specialised facilities, and it is expensive, time consuming and difficult; furthermore, it is unlikely to provide a definitive result in all cases as the expression of virulence by different strains is likely to be relative rather than absolute. To demonstrate a complete lack of virulence it is necessary to optimise the infection protocol and to include control strains that have been identified as being highly virulent and of reduced virulence, respectively. Other evidence for a lack of virulence of the strains from the multiplier herds in this and other studies would be the identification of the same strain(s) in other healthy herds that received pigs from these herds. This was not possible in this case as they all stopped supplying pigs to other herds once they were found to be carrying *B*. *hyodysenteriae*, and none of the herds that previously had received pigs reported developing SD.

If isolates from some or all of the multiplier herds from the current study were shown to cause disease in experimentally infected pigs, the question would arise as to why did pigs in these herds remain healthy? Previously an isolate of *B*. *hyodysenteriae* that was recovered from a healthy multiplier herd in Australia was shown to induce SD when used to experimentally infect pigs, and it was assumed that this might reflect differences in the gastrointestinal ecology in pigs from different sources [5]. It is known that under experimental conditions the expression of SD can be influenced by dietary ingredients that are fed to pigs: these different substrates have been shown to influence the microbiota and/or the physicochemical environment in the colon (eg viscosity, hydration, pH), which in turn may influence the ability of *B*. *hyodysenteriae* to colonise [8–12,47]. In the current study the pigs in the various herds were all fed standard commercial diets, so diet is not an obvious explanation for any differences in disease occurrence. Another difference is that in multiplier and breeding herds there is likely to be less crowding, stress and concurrent disease compared to production herds, due to their superior management practices. It is known that the hormone norepinephrine is released into the intestinal tract in times of stress, and under experimental *in vitro* conditions norepinephrine has been shown to increase proliferation and attachment to cell monolayers by the related spirochaete *Brachyspira pilosicoli* [48]: it seems likely that the same may apply to *B*. *hyodysenteriae*. The likelihood of such circumstances occurring in the multiplier herds is supported by the finding of an isolate of “*B*. *hampsonii*” in herd B, suggesting that it too may not have had an optimised colonic environment to allow it to induce disease, even though there was a heavy growth in the colon. In the case of the *B*. *hyodysenteriae* isolates, the lack of members of the block of six plasmid genes also may have reduced their colonisation fitness, which together with a lack of other predisposing factors in the multiplier herds could have resulted in a level of colonisation that was insufficient to induce signs of disease.

Unfortunately breeding and multiplier herds with pigs that do not show signs of disease but that harbour strains of *B*. *hyodysenteriae* are placed in a difficult position when it comes to selling stock. In the case of the three herds described here they ceased selling live pigs. In the current work it was not possible to definitively use the genotype to identify strains that had reduced virulence, but strain J11 in herd C had significant differences with respect to having weak haemolysis, and potentially diminished iron binding and lack of plasmid genes that suggest a reduced potential to cause disease. Strains with this sort of profile could be regarded as presenting a low risk of causing disease, although further work is required to confirm this. It is important to note that strains that lack some or all of the block of six plasmid genes still may be able to cause disease under conditions of poor husbandry, stress or dietary changes that can enhance colonisation and predispose to disease development.

## Supporting Information

S1 Table Identity of the 332 virulence-associated genes investigated for their distribution in the German *B*. *hyodysenteriae* isolates. (DOCX)

S2 Table Quality statistics for mapping the 23 strains of *B*. *hyodysenteriae* with the reference strain WA1. (DOCX)

S3 Table Sequence information and assembly statistics for the 23 sequenced *B*. *hyodysenteriae* strains. (DOCX)
