# Supplementary material for: Comparison of Brachyspira hyodysenteriae Isolates Recovered from Pigs in Apparently Healthy Multiplier Herds with Isolates from Herds with Swine Dysentery
Source: PLoS One. 2016 Aug 4;11(8):e0160362. doi: 10.1371/journal.pone.0160362 (PMC4973917; doi:10.1371/journal.pone.0160362)
Supplement: S1 Table — (DOCX) [file pone.0160362.s001.docx]

**Table S1. Identity of the 332 virulence-associated genes investigated for their distribution in the German *B. hyodysenteriae* isolates.**

| **Locus** | **Gene** | **Putative function** | **Position on WA1 genome**  **or plasmid (bp)** |
| --- | --- | --- | --- |
| BHWA1_RS00085 | *fli*W | Flagellar assembly protein | 19296 - 19772 |
| BHWA1_RS00090 | *flg*K | Flagellar hook-associated protein | 19842 - 21095 |
| BHWA1_RS00140 | na | Membrane protein | 28985 - 29752 |
| BHWA1_RS00235 | *mcp*A | Methyl-accepting chemotaxis protein | 50604 - 52442 |
| BHWA1_RS00240 | *mcp*A | Methyl-accepting chemotaxis protein | 52855 - 54696 |
| BHWA1_RS00340 | *arp* | Acid repeat protein | 81451 - 83805 |
| BHWA1_RS00380 | na | Protease | 92975 - 93499 |
| BHWA1_RS00420 | *kds*A | 3-deoxy-manno-octulosonate-8-phosphate synthetase | 101438 - 102253 |
| BHWA1_RS00500 | *arp* | Acid repeat protein | 115938 - 116696 |
| BHWA1_RS00515 | *arp* | Acid repeat protein | 118755 - 120260 |
| BHWA1_RS00560 | *arp* | Acid repeat protein | 128964 - 130112 |
| BHWA1_RS00655 | *mcp*B | Methyl-accepting chemotaxis protein | 152946 - 154880 |
| BHWA1_RS00725 | na | Peptidase | 169454 - 170695 |
| BHWA1_RS00760 | *kdt*A | 3-deoxy-manno-octulosonate transferase | 178146 - 179408 |
| BHWA1_RS00805 | *mcp* | Methyl-accepting chemotaxis protein | 185511 - 187340 |
| BHWA1_RS00890 | na | Aminopeptidase | 202278 - 203402 |
| BHWA1_RS00995 | *mcp* | Methyl-accepting chemotaxis protein | 224339 - 227005 |
| BHWA1_RS01000 | *mcp* | Methyl-accepting chemotaxis protein | 227202 - 230057 |
| BHWA1_RS01005 | *omp*A | Outer membrane protein | 230078 - 231289 |
| BHWA1_RS01015 | *arp* | Acid repeat protein | 233675 - 234421 |
| BHWA1_RS01080 | *arp* | Acid repeat protein | 244405 - 245094 |
| BHWA1_RS01085 | *arp* | Acid repeat protein | 245333 - 246586 |
| BHWA1_RS01170 | *tly*A | Haemolysin | 267198 - 267920 |
| BHWA1_RS01220 | *arp* | Acid repeat protein | 280976 - 282358 |
| BHWA1_RS01225 | *arp* | Acid repeat protein | 282439 - 285075 |
| BHWA1_RS01260 | na | Membrane protein | 291406 - 292332 |
| BHWA1_RS01310 | na | Peptidase | 301576 - 302964 |
| BHWA1_RS01315 | na | Peptidase | 302965 - 304320 |
| BHWA1_RS01415 | *lpx*L | lauryl-acyl carrier protein (ACP)-dependent acyltransferase | 323282 - 324244 |
| BHWA1_RS01425 | *lol*A | Outer membrane protein | 324925 - 325494 |
| BHWA1_RS01430 | na | Membrane protein | 325487 - 327793 |
| BHWA1_RS01450 | *arp* | Acid repeat protein | 330371 - 331039 |
| BHWA1_RS01485 | *arp* | Acid repeat protein | 337094 - 338719 |
| BHWA1_RS01640 | na | Membrane protein | 368141 - 369112 |
| BHWA1_RS01670 | na | Peptidase | 374823 - 377225 |
| BHWA1_RS01685 | *fli*P | Flagellar biosynthesis protein | 379929 - 380717 |
| BHWA1_RS01690 | *fli*Q | Flagellar biosynthesis protein | 380770 - 381039 |
| BHWA1_RS01695 | *fli*R | Flagellar biosynthesis protein | 381089 - 381892 |
| BHWA1_RS01700 | *flh*B | Flagellar biosynthesis protein | 381889 - 383091 |
| BHWA1_RS01705 | *flh*A | Flagellar biosynthesis protein | 383113 - 385224 |
| BHWA1_RS01715 | *fle*N | Flagellar synthesis regulator | 387587 - 388450 |
| BHWA1_RS01715 | *flh*F | Flagellar biosynthesis protein | 387587 - 388450 |
| BHWA1_RS01765 | *arp* | Acid repeat protein | 398392 - 399549 |
| BHWA1_RS01795 | na | Peptidase | 407487 - 408314 |
| BHWA1_RS01805 | na | Peptidase | 409556 - 410740 |
| BHWA1_RS01815 | na | Phospholipase | 411934 - 412830 |
| BHWA1_RS01825 | na | Papain-like cysteine peptidase | 414597 - 415460 |
| BHWA1_RS01845 | na | Membrane protein | 419476 - 422256 |
| BHWA1_RS01905 | *arp* | Acid repeat protein | 431435 - 431779 |
| BHWA1_RS01915 | na | Outer membrane protein | 433649 - 435595 |
| BHWA1_RS02000 | *arp* | Acid repeat protein | 456035 - 458005 |
| BHWA1_RS02045 | *mcp*B | Methyl-accepting chemotaxis protein | 466817 - 468670 |
| BHWA1_RS02080 | *mcp* | Methyl-accepting chemotaxis protein | 475388 - 477205 |
| BHWA1_RS02180 | na | Protease | 499600 - 500298 |
| BHWA1_RS02185 | *mcp*B | Methyl-accepting chemotaxis protein | 500447 - 502558 |
| BHWA1_RS02195 | *ypl*Q | Haemolysin III | 503722 - 504396 |
| BHWA1_RS02280 | *mcp*C | Methyl-accepting chemotaxis protein | 549938 - 551689 |
| BHWA1_RS02310 | *glm*U | N-acetylglucosamine-1-phosphate uridyltransferase | 558549 - 560084 |
| BHWA1_RS02360 | *arp* | Acid repeat protein | 570910 - 571281 |
| BHWA1_RS02365 | *arp* | Acid repeat protein | 571396 - 571713 |
| BHWA1_RS02370 | *arp* | Acid repeat protein | 571930 - 572628 |
| BHWA1_RS02395 | *mcp* | Methyl-accepting chemotaxis protein | 577312 - 579240 |
| BHWA1_RS02405 | *che*Y | Chemosensory transduction protein | 579548 - 579916 |
| BHWA1_RS02410 | *che*A | Chemosensory transduction protein | 579969 - 582131 |
| BHWA1_RS02415 | *che*W | Chemosensory transduction protein | 582143 - 582673 |
| BHWA1_RS02420 | *che*R | Chemosensory transduction protein | 582692 - 583543 |
| BHWA1_RS02425 | *che*D | Chemosensory transduction protein | 583560 - 584153 |
| BHWA1_RS02430 | *che*B | Chemosensory transduction protein | 584178 - 585311 |
| BHWA1_RS02490 | na | Outer membrane protein | 599574 - 601079 |
| BHWA1_RS02555 | na | Peptidase | 617363 - 618802 |
| BHWA1_RS02565 | *mcp* | Methyl-accepting chemotaxis protein | 620761 - 622593 |
| BHWA1_RS02690 | *mcp*B | Methyl-accepting chemotaxis protein | 644234 - 646498 |
| BHWA1_RS02695 | *mcp*B | Methyl-accepting chemotaxis protein | 646721 - 648955 |
| BHWA1_RS02700 | *fla*A | Flagellar filament outer layer protein | 649023 - 649826 |
| BHWA1_RS02800 | na | Membrane protein | 675358 - 678348 |
| BHWA1_RS02825 | na | Membrane protein | 684641 - 687646 |
| BHWA1_RS02830 | na | Membrane protein | 688008 - 691001 |
| BHWA1_RS02875 | *che*X | Chemosensory transduction protein | 703594 - 704094 |
| BHWA1_RS02875 | *che*X | Chemosensory transduction protein | 703654 - 704094 |
| BHWA1_RS02885 | *hly* | Haemolysin activation protein | 704803 - 706191 |
| BHWA1_RS02900 | *arp* | Acid repeat protein | 708468 - 709706 |
| BHWA1_RS02905 | *arp* | Acid repeat protein | 709767 - 710864 |
| BHWA1_RS02910 | na | Membrane protein | 710896 - 712146 |
| BHWA1_RS02920 | *arp* | Acid repeat protein | 714145 - 714729 |
| BHWA1_RS02925 | *arp* | Acid repeat protein | 715385 - 716473 |
| BHWA1_RS02935 | *arp* | Acid repeat protein | 717806 - 718846 |
| BHWA1_RS02985 | na | Peptidase | 729957 - 731777 |
| BHWA1_RS03035 | *pep*P | Aminopeptidase | 741898 - 743013 |
| BHWA1_RS03265 | *fli*I | Flagellar biosynthesis protein | 795318 - 796772 |
| BHWA1_RS03270 | *fli*H | Flagellar assembly protein | 796813 - 797715 |
| BHWA1_RS03275 | *fli*G | Flagellar motor switch protein | 797757 - 798788 |
| BHWA1_RS03280 | *fli*F | Flagellar MS-ring protein | 798848 - 800476 |
| BHWA1_RS03395 | na | Peptidase | 820294 - 821355 |
| BHWA1_RS03415 | *ftn*A | Ferritin | 823727 - 824269 |
| BHWA1_RS03435 | na | Patatin-like phospholipase | 831342 - 832109 |
| BHWA1_RS03440 | *fli*D | Flagellar hook-associated protein | 832194 - 834395 |
| BHWA1_RS03470 | *yng*K | Phospholipase | 838583 - 840400 |
| BHWA1_RS03510 | *che*W | Chemosensory transduction protein | 846511 - 846990 |
| BHWA1_RS03535 | *pep*P | Aminopeptidase | 852744 - 853820 |
| BHWA1_RS03540 | na | Membrane protein | 853897 - 854787 |
| BHWA1_RS03580 | *mcp*C | Methyl-accepting chemotaxis protein | 860308 - 861540 |
| BHWA1_RS03600 | *arp* | Acid repeat protein | 865013 - 866638 |
| BHWA1_RS03760 | *clp*P | Periplasmic serine protease | 915159 - 916010 |
| BHWA1_RS03895 | *arp* | Acid repeat protein | 947215 - 947856 |
| BHWA1_RS03950 | na | Membrane protein | 960702 - 961502 |
| BHWA1_RS04120 | *fli*E | Flagellar hook-basal body protein | 993004 - 993375 |
| BHWA1_RS04125 | *flg*C | Flagellar basal body rod protein | 993910 - 994338 |
| BHWA1_RS04130 | *flg*B | Flagellar basal body rod protein | 993910 - 994338 |
| BHWA1_RS04155 | na | Metallo-endopeptidase | 998081 - 999322 |
| BHWA1_RS04180 | *arp* | Acid repeat protein | 1005472 - 1007139 |
| BHWA1_RS04210 | na | Membrane protein | 1015452 - 1016309 |
| BHWA1_RS04220 | na | Membrane protein | 1016744 - 1017730 |
| BHWA1_RS04245 | *bit*A | Periplasmic iron-binding protein | 1021776 - 1022747 |
| BHWA1_RS04250 | *bit*B | Periplasmic iron-binding protein | 1022782 - 1023810 |
| BHWA1_RS04255 | *bit*C | Periplasmic iron-binding protein | 1023891 - 1024910 |
| BHWA1_RS04320 | *kds*C | 3-deoxy-manno-octulosonate-8-phosphate phosphatase | 1041225 - 1041776 |
| BHWA1_RS04355 | *bhmp39a* | Outer membrane protein | 1046353 - 1047399 |
| BHWA1_RS04395 | na | Membrane protein | 1052587 - 1053219 |
| BHWA1_RS04410 | na | Peptidase | 1055536 - 1057548 |
| BHWA1_RS04485 | *flg*G | Flagellar basal body rod protein | 1071159 - 1071953 |
| BHWA1_RS04515 | na | Membrane protein | 1082897 - 1083580 |
| BHWA1_RS04705 | na | Haemolysin | 1121381 - 1122679 |
| BHWA1_RS04780 | *mcp*A | Methyl-accepting chemotaxis protein | 1142948 - 1144876 |
| BHWA1_RS04780 | *mcp*A | Methyl-accepting chemotaxis protein | 950495 - 951508 |
| BHWA1_RS04785 | *arp* | Acid repeat protein | 1145528 - 1147660 |
| BHWA1_RS04790 | *arp* | Acid repeat protein | 1147838 - 1149982 |
| BHWA1_RS04795 | *mcp* | Flagellar filament outer layer protein | 1151599 - 1151976 |
| BHWA1_RS04885 | *che*Y | Chemosensory transduction protein | 1166793 - 1167218 |
| BHWA1_RS04945 | *mcp* | Methyl-accepting chemotaxis protein | 1183505 - 1184827 |
| BHWA1_RS04955 | *mcp*B | Methyl-accepting chemotaxis protein | 1186287 - 1188236 |
| BHWA1_RS04960 | *tar*5 | Methyl-accepting chemotaxis protein | 1188451 - 1190367 |
| BHWA1_RS04965 | *mcp*B | Methyl-accepting chemotaxis protein | 1190674 - 1191786 |
| BHWA1_RS04965 | *mcp*B | Methyl-accepting chemotaxis protein | 1190674 - 1191786 |
| BHWA1_RS05010 | na | Membrane protein | 1205109 - 1206194 |
| BHWA1_RS05055 | *arp* | Acid repeat protein | 1214969 - 1216552 |
| BHWA1_RS05135 | *tmp*B | Outer membrane protein | 1231562 - 1232980 |
| BHWA1_RS05140 | na | Outer membrane protein | 1233070 - 1233660 |
| BHWA1_RS05150 | *arp* | Acid repeat protein | 1234440 - 1234892 |
| BHWA1_RS05155 | *arp* | Acid repeat protein | 1235031 - 1235300 |
| BHWA1_RS05160 | *arp* | Acid repeat protein | 1235483 - 1235917 |
| BHWA1_RS05165 | *arp* | Acid repeat protein | 1235953 - 1236408 |
| BHWA1_RS05200 | *bhlp*16 | Outer membrane protein | 1246414 - 1246917 |
| BHWA1_RS05330 | *arp* | Acid repeat protein | 1274737 - 1275879 |
| BHWA1_RS05440 | na | Outer membrane protein | 1296310 - 1296702 |
| BHWA1_RS05460 | *arp* | Acid repeat protein | 1300607 - 1301701 |
| BHWA1_RS05470 | *arp* | Acid repeat protein | 1303660 - 1304805 |
| BHWA1_RS05555 | *glm*S | Glucosamine-6-phosphate synthase | 1323858 - 1325684 |
| BHWA1_RS05595 | *mcp*B | Methyl-accepting chemotaxis protein | 1334216 - 1335877 |
| BHWA1_RS05965 | *tly*B | Haemolysin | 1414869 - 1417355 |
| BHWA1_RS06040 | *fla*A | Flagellar filament outer layer protein | 1429907 - 1430869 |
| BHWA1_RS06100 | na | Membrane protein | 1439949 - 1440803 |
| BHWA1_RS06115 | *arp* | Acid repeat protein | 1442969 - 1443556 |
| BHWA1_RS06140 | *lpx*C | UDP-3-O-acyl-N-acetylglucosamine deacetylase | 1447436 - 1448740 |
| BHWA1_RS06215 | *mcp*B | Methyl-accepting chemotaxis protein | 1466100 - 1467911 |
| BHWA1_RS06225 | *mcp*A | Methyl-accepting chemotaxis protein | 1468688 - 1470535 |
| BHWA1_RS06230 | *mcp*A | Methyl-accepting chemotaxis protein | 1470821 - 1472668 |
| BHWA1_RS06320 | *mcp*B | Methyl-accepting chemotaxis protein | 1494123 - 1495949 |
| BHWA1_RS06440 | na | Membrane protein | 1532143 - 1532877 |
| BHWA1_RS06470 | *che*X | Chemosensory transduction protein | 1542101 - 1542568 |
| BHWA1_RS06475 | *che*Y | Chemosensory transduction protein | 1542575 - 1542994 |
| BHWA1_RS06480 | *che*R | Chemosensory transduction protein | 1543029 - 1543844 |
| BHWA1_RS06535 | *fts*H | ATP-dependent metalloprotease | 1551671 - 1553767 |
| BHWA1_RS06560 | *mcp*A | Methyl-accepting chemotaxis protein | 1557385 - 1559220 |
| BHWA1_RS06620 | *pld*B | Lysophospholipase | 1571615 - 1572547 |
| BHWA1_RS06660 | na | Outer membrane protein | 1581918 - 1584554 |
| BHWA1_RS06665 | *omp*H | Outer membrane protein | 1584599 - 1585024 |
| BHWA1_RS06670 | *lpx*D | UDP-3-O-acyl-N-acetylglucosamine N-acyltransferase | 1585133 - 1586173 |
| BHWA1_RS06675 | *mcp*C | Methyl-accepting chemotaxis protein | 1586290 - 1588254 |
| BHWA1_RS06735 | na | Peptidase | 1598366 - 1599547 |
| BHWA1_RS06745 | *fli*N | Flagellar motor switch protein | 1599939 - 1600193 |
| BHWA1_RS06750 | *fli*N | Flagellar motor switch protein | 1600390 - 1601472 |
| BHWA1_RS06755 | *fli*M | Flagellar motor switch protein | 1601477 - 1602508 |
| BHWA1_RS06760 | *fli*L | Flagellar basal body-associated protein | 1602555 - 1603100 |
| BHWA1_RS06780 | *che*C | Chemosensory transduction protein | 1613366 - 1613839 |
| BHWA1_RS06805 | *che*Y1 | Chemosensory transduction protein | 1616729 - 1617085 |
| BHWA1_RS06830 | na | Membrane protein | 1625635 - 1625898 |
| BHWA1_RS06835 | na | Aminopeptidase | 1625911 - 1626708 |
| BHWA1_RS06870 | *mot*B | Flagellar motor protein | 1633392 - 1634192 |
| BHWA1_RS06875 | *mot*A | Flagellar motor protein | 1634211 - 1635002 |
| BHWA1_RS06880 | *flb*D | Flagellar family protein | 1635042 - 1635203 |
| BHWA1_RS06885 | *flg*E | Flagellar hook protein | 1635333 - 1636604 |
| BHWA1_RS06905 | na | Peptidase | 1640362 - 1641261 |
| BHWA1_RS06915 | *arp* | Acid repeat protein | 1642387 - 1643076 |
| BHWA1_RS06925 | *tly*C | Haemolysin | 1644980 - 1645786 |
| BHWA1_RS07045 | *fla*B3 | Periplasmic flagellar filament protein | 1673068 - 1673838 |
| BHWA1_RS07050 | na | Aminopeptidase | 1674206 - 1675195 |
| BHWA1_RS07055 | na | Zn dependant metalloprotease | 1675558 - 1676268 |
| BHWA1_RS07060 | *bhmp*39d | Outer membrane protein | 1676363 - 1677499 |
| BHWA1_RS07065 | *bhmp*39c | Outer membrane protein | 1677675 - 1678823 |
| BHWA1_RS07070 | *bhmp*39b | Outer membrane protein | 1678996 - 1680093 |
| BHWA1_RS07075 | *bhmp*39d | Outer membrane protein | 1680314 - 1680496 |
| BHWA1_RS07135 | na | Aminopeptidase | 1694325 - 1695758 |
| BHWA1_RS07160 | *lpx*M | Myristoyl-acyl carrier protein (ACP)-dependent acyltransferase | 1700225 - 1701130 |
| BHWA1_RS07165 | na | Protease | 1701396 - 1702907 |
| BHWA1_RS07280 | *fla*B | Periplasmic flagellar filament protein | 1728809 - 1729588 |
| BHWA1_RS07285 | na | Membrane protein | 1730697 - 1731302 |
| BHWA1_RS07470 | na | Membrane protein | 1775264 - 1777138 |
| BHWA1_RS07525 | na | Outer membrane protein | 1785123 - 1786205 |
| BHWA1_RS07530 | na | Outer membrane protein | 1786252 - 1787325 |
| BHWA1_RS07535 | na | Outer membrane protein | 1787339 - 1788412 |
| BHWA1_RS07540 | na | Outer membrane protein | 1788520 - 1789548 |
| BHWA1_RS07555 | na | Membrane protein | 1792295 - 1793410 |
| BHWA1_RS07560 | na | Membrane protein | 1793442 - 1794395 |
| BHWA1_RS07600 | na | Peptidase | 1800347 - 1801084 |
| BHWA1_RS07695 | *mcp*A | Methyl-accepting chemotaxis protein | 1819716 - 1821560 |
| BHWA1_RS07730 | *mcp*B | Methyl-accepting chemotaxis protein | 1830257 - 1832098 |
| BHWA1_RS07785 | *bhmp*39e | Outer membrane protein | 1841415 - 1842575 |
| BHWA1_RS07790 | *bhmp*39f | Membrane protein | 1842946 - 1844079 |
| BHWA1_RS07810 | na | Aminopeptidase | 1848749 - 1849555 |
| BHWA1_RS07835 | na | Membrane protein | 1854323 - 1855702 |
| BHWA1_RS07910 | *mcp*A | Methyl-accepting chemotaxis protein | 1875329 - 1877179 |
| BHWA1_RS07990 | *che*D2 | Chemosensory transduction protein | 1892904 - 1894718 |
| BHWA1_RS08025 | na | Membrane protein | 1901656 - 1903005 |
| BHWA1_RS08035 | *mcp*B | Methyl-accepting chemotaxis protein | 1905156 - 1907993 |
| BHWA1_RS08040 | *flg*K | Flagellar hook-associated protein | 1908183 - 1910201 |
| BHWA1_RS08055 | *mcp*B | Methyl-accepting chemotaxis protein | 1912287 - 1914113 |
| BHWA1_RS08060 | *mcp*B | Methyl-accepting chemotaxis protein | 1914329 - 1916170 |
| BHWA1_RS08275 | na | Membrane protein | 1969029 - 1969517 |
| BHWA1_RS08380 | na | ATP-dependent protease | 1989453 - 1991978 |
| BHWA1_RS08385 | *mcp*B | Methyl-accepting chemotaxis protein | 1992366 - 1994279 |
| BHWA1_RS08395 | na | Peptidase | 1995761 - 1996954 |
| BHWA1_RS08420 | *mcp* | Methyl-accepting chemotaxis protein | 2002640 - 2005264 |
| BHWA1_RS08445 | na | Membrane protein | 2010374 - 2011021 |
| BHWA1_RS08510 | na | Membrane protein | 2029216 - 2029896 |
| BHWA1_RS08520 | *bhlp*29.7b | Outer membrane protein | 2030898 - 2031695 |
| BHWA1_RS08525 | *bhlp*29.7f | Outer membrane protein | 2031727 - 2032515 |
| BHWA1_RS08530 | *bhlp*29.7e | Outer membrane protein | 2032536 - 2033324 |
| BHWA1_RS08535 | *bhmp*29.7 | Membrane protein | 2033403 - 2034218 |
| BHWA1_RS08580 | *nfe*D | Membrane-bound serine protease | 2040746 - 2042080 |
| BHWA1_RS08585 | na | Membrane protein | 2042616 - 2043053 |
| BHWA1_RS08660 | na | Membrane protein | 2057710 - 2058585 |
| BHWA1_RS08705 | na | Outer membrane protein | 2067234 - 2067845 |
| BHWA1_RS08715 | *arp* | Acid repeat protein | 2069854 - 2070549 |
| BHWA1_RS08770 | na | Membrane protein | 2082472 - 2082972 |
| BHWA1_RS08825 | na | Membrane protein | 2093237 - 2093749 |
| BHWA1_RS08870 | *mcp*B | Methyl-accepting chemotaxis protein | 2105643 - 2107847 |
| BHWA1_RS09020 | *mcp*B | Methyl-accepting chemotaxis protein | 2138169 - 2140007 |
| BHWA1_RS09025 | *mcp*B | Methyl-accepting chemotaxis protein | 2140227 - 2142077 |
| BHWA1_RS09070 | na | Peptidase | 2151374 - 2153551 |
| BHWA1_RS09085 | na | Haemolysin III channel protein | 2156246 - 2156917 |
| BHWA1_RS09115 | na | Protease | 2162055 - 2163524 |
| BHWA1_RS09170 | *kds*B | 3-deoxy-manno-octulosonate cytidylyltransferase | 2175924 - 2176694 |
| BHWA1_RS09215 | *arp* | Acid repeat protein | 2182788 - 2183894 |
| BHWA1_RS09410 | *mcp*B | Methyl-accepting chemotaxis protein | 2227816 - 2229561 |
| BHWA1_RS09410 | *mcp*B | Methyl-accepting chemotaxis protein | 2227816 - 2229561 |
| BHWA1_RS09425 | na | Peptidase | 2233861 - 2235087 |
| BHWA1_RS09480 | na | Membrane protein | 2245882 - 2246838 |
| BHWA1_RS09505 | *mcp* | Methyl-accepting chemotaxis protein | 2252065 - 2253750 |
| BHWA1_RS09605 | na | Membrane protein | 2271759 - 2272532 |
| BHWA1_RS09610 | na | Membrane protein | 2273053 - 2273886 |
| BHWA1_RS09730 | *flg*D | Flagellar hook capping protein | 2302198 - 2302992 |
| BHWA1_RS09735 | *fli*K | Flagellar hook-length control protein | 2303043 - 2304650 |
| BHWA1_RS09785 | na | Membrane protein | 2312838 - 2313038 |
| BHWA1_RS09810 | *nox* | NADH oxidase | 2315814 - 2317154 |
| BHWA1_RS09880 | *arp* | Acid repeat protein | 2330569 - 2331579 |
| BHWA1_RS09885 | *arp* | Acid repeat protein | 2332020 - 2333312 |
| BHWA1_RS09890 | *arp* | Acid repeat protein | 2334028 - 2335032 |
| BHWA1_RS09900 | na | Outer membrane protein | 2338446 - 2339843 |
| BHWA1_RS09905 | na | Membrane protein | 2339851 - 2341110 |
| BHWA1_RS09945 | na | Zn-dependent peptidase | 2350267 - 2351532 |
| BHWA1_RS10110 | na | Membrane protein | 2387303 - 2390005 |
| BHWA1_RS10210 | na | Peptidase | 2408508 - 2409464 |
| BHWA1_RS10235 | na | Membrane-associated zinc metalloprotease | 2415027 - 2416337 |
| BHWA1_RS10265 | na | Outer membrane protein | 2421783 - 2422514 |
| BHWA1_RS10485 | *lpx*B | tetra-acyl-disaccharide-1-phosphate synthase | 2449587 - 2450717 |
| BHWA1_RS10490 | *lpx*A | UDP-N-acetylglucosamine acyltransferase | 2450730 - 2451524 |
| BHWA1_RS10530 | *arp* | Acid repeat protein | 2459889 - 2461883 |
| BHWA1_RS10545 | *clp*YQ | ATP-dependent protease | 2462887 - 2463417 |
| BHWA1_RS10550 | *clp*YQ | ATP-dependent protease | 2463420 - 2464781 |
| BHWA1_RS10565 | *che*B | Chemosensory transduction protein | 2467736 - 2468905 |
| BHWA1_RS10645 | *tol*C | Outer membrane protein | 2483120 - 2484574 |
| BHWA1_RS10695 | *arp* | Acid repeat protein | 2492224 - 2492976 |
| BHWA1_RS10700 | *arp* | Acid repeat protein | 2493324 - 2494085 |
| BHWA1_RS10710 | *arp* | Acid repeat protein | 2495311 - 2496021 |
| BHWA1_RS10740 | *mcp* | Methyl-accepting chemotaxis protein | 2502587 - 2504305 |
| BHWA1_RS10820 | na | ATP-dependent Clp protease | 2522484 - 2523080 |
| BHWA1_RS10825 | *clp*X | ATP-dependent Clp protease | 2523262 - 2524494 |
| BHWA1_RS11040 | na | Aminopeptidase | 2571061 - 2571855 |
| BHWA1_RS11065 | *arp* | Acid repeat protein | 2575872 - 2576849 |
| BHWA1_RS11100 | *che*W | Chemosensory transduction protein | 2584938 - 2585453 |
| BHWA1_RS11100 | *che*W | Chemosensory transduction protein | 2585055 - 2585453 |
| BHWA1_RS11205 | *mcp* | Methyl-accepting chemotaxis protein | 2605710 - 2607527 |
| BHWA1_RS11210 | *mcp* | Methyl-accepting chemotaxis protein | 2607534 - 2609354 |
| BHWA1_RS11215 | *lys*M | Peptidase | 2609466 - 2611283 |
| BHWA1_RS11365 | *fla*A | Flagellar filament outer layer protein | 2642375 - 2643208 |
| BHWA1_RS11370 | *fla*A | Flagellar filament outer layer protein | 2643234 - 2643938 |
| BHWA1_RS11390 | *arp* | Acid repeat protein | 2646190 - 2646618 |
| BHWA1_RS11445 | na | Protease | 2658157 - 2658819 |
| BHWA1_RS11460 | na | Membrane protein | 2660796 - 2661512 |
| BHWA1_RS11545 | *nag*B | Glucosamine-6-phosphate deaminase | 2679057 - 2679818 |
| BHWA1_RS11565 | *vsp*H | Membrane protein | 2683159 - 2684469 |
| BHWA1_RS11810 | na | Peptidase | 2743496 - 2744101 |
| BHWA1_RS11825 | na | Metal-dependent carboxypeptidase | 2748985 - 2750211 |
| BHWA1_RS11850 | *arp* | Acid repeat protein | 2753760 - 2754173 |
| BHWA1_RS11885 | *arp* | Acid repeat protein | 2760419 - 2760994 |
| BHWA1_RS12030 | *mcp* | Methyl-accepting chemotaxis protein | 2786231 - 2788207 |
| BHWA1_RS12035 | *mcp* | Methyl-accepting chemotaxis protein | 2788219 - 2790153 |
| BHWA1_RS12080 | na | Membrane dipeptidase | 2800735 - 2801685 |
| BHWA1_RS12090 | na | Membrane protein | 2802437 - 2803003 |
| BHWA1_RS12095 | *nox* | NADH oxidase | 2803154 - 2804512 |
| BHWA1_RS12350 | *che*A | Chemosensory transduction protein | 2864792 - 2867182 |
| BHWA1_RS12385 | na | Membrane protein | 2873632 - 2874654 |
| BHWA1_RS12395 | *bplp*35 | D-galactose-binding periplasmic protein | 2876298 - 2877350 |
| BHWA1_RS12400 | *bplp*35 | D-galactose-binding periplasmic protein | 2877669 - 2878697 |
| BHWA1_RS12405 | *bplp*35 | D-galactose-binding periplasmic protein | 2878783 - 2879799 |
| BHWA1_RS12475 | na | Membrane protein | 2892263 - 2893927 |
| BHWA1_RS12535 | na | Membrane protein | 2906692 - 2908305 |
| BHWA1_RS12540 | *mcp* | Methyl-accepting chemotaxis protein | 2908386 - 2911280 |
| BHWA1_RS12595 | na | Membrane protein | 2926593 - 2927711 |
| BHWA1_RS12665 | na | Membrane protein | 2939710 - 2940753 |
| BHWA1_RS12750 | *fli*S | Flagellar protein | 2961621 - 2962097 |
| BHWA1_RS12770 | *nag*A | N-acetylglucosamine-6-phosphate deacetylase | 2963925 - 2965889 |
| BHWA1_RS12775 | *nag*B | Glucosamine-6-phosphate deaminase | 2965903 - 2966676 |
| BHWA1_RS12830 | *hly*A | Haemolysin | 2978621 - 2978857 |
| BHWA1_RS12955 | na | Glycosyl transferase | 2068 - 2988 ^b^ |
| BHWA1_RS12960 | na | Glycosyl transferase | 3013 - 4014 ^b^ |
| BHWA1_RS12965 | na | Glycosyl transferase | 4023 - 5051 ^b^ |
| BHWA1_RS12970 | na | Glycosyl transferase | 5071 - 6900 ^b^ |
| BHWA1_RS13000 | na | Fe-S oxidoreductase containing radical SAM domain | 12844 - 14064 ^b^ |
| BHWA1_RS13005 | na | Fe-S oxidoreductase containing radical SAM domain | 14069 - 15070 ^b^ |
| BHWA1_RS13015 | na | Fe-S oxidoreductase containing radical SAM domain | 15095 - 16105 ^b^ |
| BHWA1_RS13015 | na | Glycosyl transferase | 16246 - 17511 ^b^ |
| BHWA1_RS13020 | na | NAD dependant epimerase | 17508 - 18368 ^b^ |
| BHWA1_RS13025 | *rfb*C | dTDP-4-dehydrorhamnose 3,5-epimerase | 18371 - 18904 ^b^ |
| BHWA1_RS13035 | *rfb*F | Glucose-1-phosphate cytidylyltransferase | 20047 - 20832 ^b^ |
| BHWA1_RS13070 | na | Alpha-1,2-fucosyltransferase | 26872 - 28743 ^b^ |
| BHWA1_RS13080 | *rfb*B | dTDP-glucose 4,6-dehydratase | 29816 - 30886 ^b^ |
| BHWA1_RS13085 | *rfb*A | Glucose-1-phosphate thymidylyltransferase | 30900 - 31763 ^b^ |
| BHWA1_RS13090 | *rfb*D | dTDP-4-keto-L-rhamnose reductase | 31782 - 32642 ^b^ |
| BHWA1_RS13095 | *rfb*C | dTDP-4-dehydrorhamnose 3,5-epimerase | 32648 - 33226 ^b^ |
| BHWA1_RS13105 | na | Glycosyl transferase | 34646 - 35770 ^b^ |
| DQ223651 ^c^ | *bhlp*17.6 | Outer membrane protein | not present |

^a^ position on the WA1 chromosome (accession number NC_012225)

^b^ position on the WA1 plasmid (accession number NC_012226)

^c^ accession number for gene not present in reference strain WA1

na, not available
