# Supplementary material for: Comparison of Brachyspira hyodysenteriae Isolates Recovered from Pigs in Apparently Healthy Multiplier Herds with Isolates from Herds with Swine Dysentery
Source: PLoS One. 2016 Aug 4;11(8):e0160362. doi: 10.1371/journal.pone.0160362 (PMC4973917; doi:10.1371/journal.pone.0160362)
Supplement: S2 Table — (DOCX) [file pone.0160362.s002.docx]

**Table S2. Quality statistics for mapping the 23 strains of *B. hyodysenteriae* with the reference strain WA1.**

| **Isolate** | **Length of sequence aligned to reference (bp)** | **Average identity of aligned sequence to the reference (%)** | **Percentage of aligned contigs** | **GC content (%)** |
| --- | --- | --- | --- | --- |
| JR1 | 2,998,584 | 94.91 | 5.45 | 27.1 |
| JR2 | 2,986,367 | 94.04 | 6.56 | 27.1 |
| JR3 | 2,977,115 | 96.18 | 10.67 | 27.1 |
| JR4 | 2,973,443 | 96.44 | 6.84 | 27.1 |
| JR5 | 3,002,458 | 96.22 | 2.59 | 27.0 |
| JR6 | 2,946,423 | 95.66 | 18.20 | 27.2 |
| JR7 | 3,058,664 | 95.16 | 3.89 | 27.0 |
| JR8 | 3,101,266 | 96.69 | 3.14 | 27.1 |
| JR9 | 2,990,962 | 96.98 | 12.93 | 27.1 |
| JR10 | 3,045,918 | 94.56 | 2.67 | 27.0 |
| JR11 | 3,037,667 | 94.21 | 4.11 | 27.1 |
| JR12 | 2,980,152 | 95.28 | 6.15 | 27.1 |
| JR13 | 2,970,973 | 94.64 | 3.28 | 27.1 |
| JR19 | 2,985,206 | 96.6 | 6.52 | 27.1 |
| JR20 | 3,080,477 | 95.86 | 2.42 | 27.0 |
| JR21 | 3,077,210 | 96.64 | 3.70 | 27.0 |
| JR23 | 3,073,512 | 96.27 | 2.28 | 27.0 |
| JR24 | 2,962,870 | 96.85 | 5.73 | 27.1 |
| JR25 | 3,056,402 | 95.96 | 0.63 | 27.0 |
| JR27 | 3,036,676 | 95.77 | 2.82 | 27.1 |
| JR36 | 2,998,717 | 95.73 | 2.73 | 27.1 |
| JR37 | 3,008,587 | 95.43 | 3.92 | 27.0 |
| JR38 | 2,848,598 | 96.78 | 8.79 | 27.1 |
