# Supplementary material for: Comparison of Brachyspira hyodysenteriae Isolates Recovered from Pigs in Apparently Healthy Multiplier Herds with Isolates from Herds with Swine Dysentery
Source: PLoS One. 2016 Aug 4;11(8):e0160362. doi: 10.1371/journal.pone.0160362 (PMC4973917; doi:10.1371/journal.pone.0160362)
Supplement: S3 Table — (DOCX) [file pone.0160362.s003.docx]

**Table S3. Sequence information and assembly statistics for the 23 sequenced *B. hyodysenteriae* isolates.**

| **Isolate** | **n** | **Coverage** | **n:N50** | **N50** | **Min** | **Median** | **Mean** | **Max** | **Sum** |
| --- | --- | --- | --- | --- | --- | --- | --- | --- | --- |
| JR1 | 752 | 82 | 9 | 99,372 | 101 | 307 | 4,350 | 418,266 | 3,271,379 |
| JR2 | 686 | 83 | 11 | 100,830 | 101 | 324 | 4,745 | 266,715 | 3,255,082 |
| JR3 | 806 | 84 | 19 | 60,257 | 100 | 300 | 4,044 | 180,314 | 3,260,105 |
| JR4 | 1,491 | 91 | 30 | 43,423 | 100 | 287 | 2,311 | 94,501 | 3,446,325 |
| JR5 | 1,818 | 109 | 12 | 78,801 | 100 | 277 | 1,958 | 377,786 | 3,560,216 |
| JR6 | 577 | 78 | 25 | 37,122 | 101 | 337 | 5,497 | 125,313 | 3,172,065 |
| JR7 | 978 | 74 | 7 | 129,524 | 101 | 326 | 3,566 | 577,248 | 3,478,750 |
| JR8 | 1,433 | 81 | 12 | 80,805 | 100 | 313 | 2,527 | 245,851 | 3,622,295 |
| JR9 | 758 | 76 | 23 | 42,986 | 104 | 336 | 4,452 | 193,505 | 3,374,747 |
| JR10 | 1,460 | 85 | 8 | 105,060 | 100 | 278 | 2,449 | 389,836 | 3,575,830 |
| JR11 | 1,144 | 82 | 5 | 206,342 | 100 | 300 | 3,000 | 606,383 | 3,432,950 |
| JR12 | 715 | 75 | 11 | 103,300 | 102 | 328 | 4,619 | 274,789 | 3,302,980 |
| JR13 | 1,129 | 77 | 8 | 109,110 | 100 | 314 | 3.044 | 439,153 | 3,437,180 |
| JR19 | 1,502 | 72 | 31 | 34,027 | 101 | 336 | 2,399 | 122,648 | 3,604,135 |
| JR20 | 1,448 | 73 | 10 | 117,998 | 100 | 310 | 2,482 | 265,733 | 3,594,222 |
| JR21 | 1,081 | 77 | 11 | 107,788 | 100 | 278 | 3,180 | 260,058 | 3,438,480 |
| JR23 | 1,449 | 75 | 10 | 119,491 | 100 | 283 | 2,483 | 302,833 | 3,598,544 |
| JR24 | 872 | 89 | 10 | 101,464 | 100 | 321 | 3,791 | 339,052 | 3,306,362 |
| JR25 | 2,555 | 71 | 4 | 322,962 | 100 | 305 | 1,543 | 784,126 | 3,944,296 |
| JR27 | 1,453 | 80 | 10 | 117,980 | 100 | 263 | 2,398 | 267,727 | 3,484,533 |
| JR36 | 1,540 | 81 | 9 | 104,800 | 100 | 300 | 2,285 | 275,468 | 3,519,904 |
| JR37 | 1,172 | 78 | 29 | 35,057 | 100 | 283 | 2,874 | 90,020 | 3,368,420 |
| JR38 | 2,389 | 96 | 71 | 15,129 | 100 | 294 | 1,531 | 79,807 | 3,659,752 |

n, total number of contigs; N50, shortest contig length at 50% of the genome size; n:N50, number of contigs in the N50; Min, minimum contig length; Median, median contig length; Mean, mean contig length; Max, maximum contig length; Sum, sum of all contig lengths; Coverage, average read depth for contigs
